# The effects of exercise and weight loss in overweight patients with hip osteoarthritis: design of a prospective cohort study

**DOI:** 10.1186/1471-2474-10-24

**Published:** 2009-02-23

**Authors:** Nienke Paans, Inge van den Akker-Scheek, Klaas van der Meer, Sjoerd K Bulstra, Martin Stevens

**Affiliations:** 1 Department of Orthopaedic Surgery, University Medical Center Groningen, Groningen, The Netherlands; 2 Department of Orthopaedic Surgery, Martini Hospital Groningen, Groningen, The Netherlands; 3 Department of General Practice, University Medical Center Groningen, Groningen, The Netherlands

## Abstract

**Background:**

Hip osteoarthritis (OA) is recognised as a substantial source of disability, with pain and loss of function as principal symptoms. An aging society and a growing number of overweight people, which is considered a risk factor for OA, contribute to the growing number of cases of hip OA. In knee OA patients, exercise as a single treatment is proven to be very effective towards counteracting pain and physical functionality, but the combination of weight loss and exercise is demonstrated to be even more effective. Exercise as a treatment for hip OA patients is also effective, however evidence is lacking for the combination of weight loss and exercise. Consequently, the aim of this study is to get a first impression of the potential effectiveness of exercise and weight loss in overweight patients suffering from hip OA.

**Methods/Design:**

This is a prospective cohort study. Patients aged 25 or older, overweight (BMI > 25) or obese (BMI > 30), with clinical and radiographic evidence of OA of the hip and able to attend exercise sessions will be included. The intervention is an 8-month exercise and weight-loss lifestyle program. Main goal is to increase aerobic capacity, lose weight and stimulate a low-calorie and active lifestyle. Primary outcome is self-reported physical functioning. Secondary outcomes include pain, stiffness, health-related quality of life and habitual activity level. Weight loss in kilograms and percentage of fat-free mass will also be measured.

**Discussion:**

The results of this study will give a first impression of potential effectiveness of exercise and weight loss as a combination program for patients with OA of the hip. Once this program is proven to be effective it may lead to postponing the moment of total hip replacement.

**Trial Registration number:**

NTR1053

## Background

Osteoarthritis (OA) is the most common joint disorder in the world[1]. OA is recognized as a substantial source of disability with significant social and financial costs due to surgical and medical interventions and frequent absenteeism from work. OA of the lower limb is primarily concentrated in the hip and knee joint. Pain is the principal symptom of OA. At first it occurs after use of the joint, and is relieved by rest. In later stages of OA, pain may be present during rest and even sleep. Other symptoms of OA include stiffness following rest and instability of the joint[2,3].

Most recent numbers from the US (1990) show incident rates of OA of the hip of 0.5 per 1000 per year[4]. In the Netherlands the incidence in 2000 was 1.25 per 1000 per year[5], and its prevalence will increase with the aging of Western society[6,7]. In Europe, the percentage of people over 65 years was 17% in the year 2004. Although at 14% the Netherlands is still below this percentage, it is expected to increase to 24% by the year 2050[8]. For this reason, the number of people with OA of the hip in the Netherlands is expected to increase by 1.8 to 4.3% in the period 2004–2024[6]. A similar trend is seen worldwide[9,10].

An additional risk factor for OA is being overweight or obese[11]. Being overweight is defined as having a Body Mass index (BMI) of 25–30 kg/m^2^, and being obese as having a BMI of 30 kg/m^2 ^or more. An increase of overweight or obese people is seen not only in America[12] but in Europe as well [13-15]. In the Netherlands in 2007, 45.5% of adults were overweight or obese[16]. Results from the 2003–2004 National Health and Nutrition Examination Survey (NHANES) indicate that 66% of American adults are either overweight or obese[17]. In this respect, not only the number of older people contributes to the increase of patients with hip OA, but the number of overweight or obese people as well.

To date, conservative treatment modalities for OA of the lower limb have focused on pain relief and preservation of joint function[7,18]. In these modalities, modification of lifestyle factors such as physical inactivity and overweight/obesity is considered a core element[7,19]. With respect to the treatment of OA of the knee, it has been proven that modification of physical inactivity and obesity is an effective conservative treatment modality. Weight loss as a single therapy reduces symptoms of OA of the knee[20,21], and therapeutic exercise induces the same effect[7]. Combining these two treatments shows even more effect on pain and functionality in knee OA[22]. However, with respect to the treatment of OA of the hip this evidence is lacking. Previously conducted studies have focussed mainly on knee OA, or the combination of knee and hip OA, without distinguishing by joint[23]. One Cochrane review, which did distinguish by joint, found that exercise treatments designed to reduce pain and improve functioning were effective in knee OA patients, but the same conclusion could not be drawn for hip OA patients due to insufficient data[24].

The aim of this prospective cohort study is thus to get a first glimpse of the potential effectiveness of a combination program of exercise and weight loss on overweight and obese patients suffering from hip OA.

## Methods/design

### Study design

A prospective cohort study will be conducted at the department of orthopaedics of University Medical Center Groningen (UMCG) in collaboration with the Allied Health Care Center for Rheumatology Rehabilitation (AHCRR) Hilberdink. The study design, procedures and informed consent are approved by the Medical Ethics Committee of UMCG.

### Identification and recruitment of study participants

Patients aged 25 or older, with clinical and radiographic evidence of OA of the hip who are also overweight (BMI > 25) or obese (BMI > 30) will be included. A BMI of 40 will be used as the upper limit. The clinical evidence of hip OA is based on the definition determined by Altman et al. (1991): a) hip internal rotation ≥ 15°, pain with internal rotation of the hip, morning stiffness of the hip for ≤ 60 minutes, or b) hip internal rotation < 15° and hip flexion of ≤ 115°, which has a sensitivity of 86% and a specificity of 75%. The radiographic diagnosis for OA of the hip will be established by means of the Kellgren and Lawrence criteria[25], of which grade 1–3 will be included.

Exclusion will be based on conditions which prevent safe participation in an exercise program (angina pectoris, peripheral vascular disease, stroke, congestive heart failure, chronic obstructive pulmonary disease, insulin-dependent diabetes, psychiatric disease, renal disease, liver disease, active cancer other than skin cancer and anaemia); problems of the foot or ankle that could interfere with an exercise program; rheumatic arthritis; an inability to walk without a cane or other assistive device; participation in another research study; inability to finish the study or unlikely to be compliant with the opinion of the clinical staff because of frailty or illness; inability to fill in a questionnaire as a result of language problems or dementia. The assessment to include or exclude will be determined by the orthopaedic specialist or the general practitioner.

**Figure 1:**
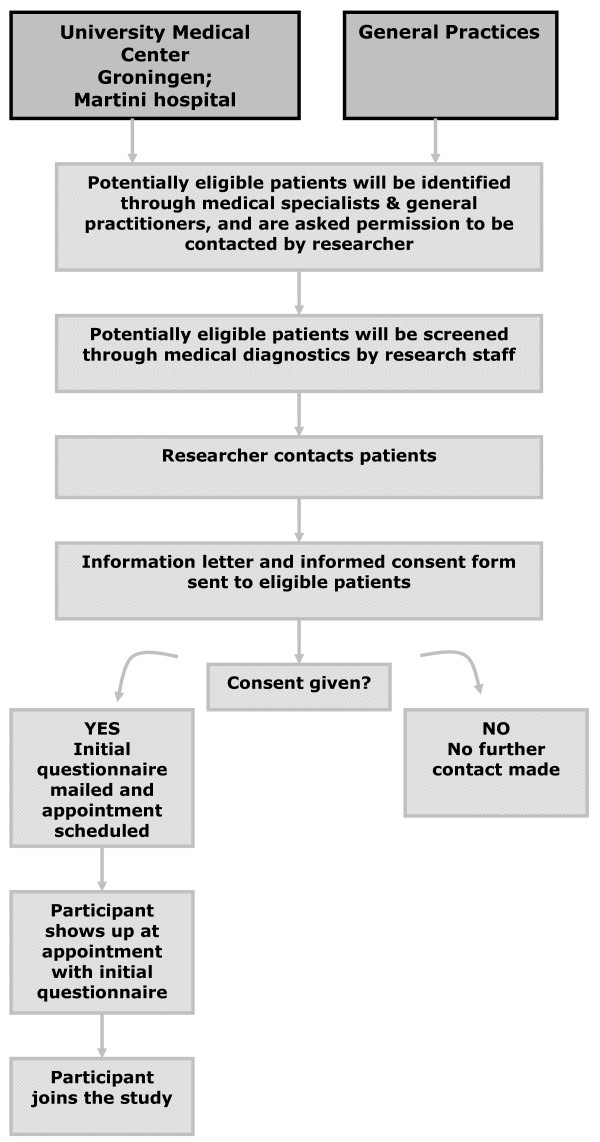
**Identification and recruitment of study participants**.

Recruitment will originate from three sources: 1) the outpatient OA clinic of the Orthopaedic Department of UMCG or the Orthopaedic Department of Martini Hospital Groningen; 2) general practices in the local area of the AHCRR and at the Department of General Practice of UMCG; and 3) patients who present themselves directly at the AHCRR and meet the inclusion criteria, as established by their general practitioner (see figure 1). Patients with hip OA who meet the inclusion criteria and are not yet indicated for hip replacement are invited to participate.

### Intervention

The intervention is an 8-month exercise and weight-loss combination program under the supervision of physiotherapists and a dietician at the AHCRR, and will be presented to the patient as a lifestyle program.

The exercise portion consists of an individual 3-month part and a 5-month group session part. The individual part consists of defining and improving the physical load potential of the patient, reducing current disabilities like lack of joint mobility and stability, optimising quality of movement, improving illness perceptions and enhancing physical fitness. The group part is focused on teaching self-management and coping, stimulating an active lifestyle, finding an optimal balance between exertion and relaxation, increasing aerobic capacity and physical fitness, increasing muscle strength, and decreasing limitations of activities of daily living. Aerobic capacity and physical fitness improvement will be achieved with the help of various devices like treadmills, free weight benches, stationary exercise bikes, steppers and/or rowing machines. All exercises will focus on personal needs, and personal preferences for aerobic equipment will be taken into consideration. A weekly exercise session lasts approximately 1 hour. In addition, patients are urged to achieve a minimum of 30 minutes of moderately intense physical activity on most, preferably all days of the week, in order to comply with national/international physical activity guidelines [26-28]. At the beginning of every exercise session patients are asked for their activities of last week.

Parallel to the individual and group phase of the exercise program, the weight-loss program is implemented by a certified dietician. This diet part of the intervention is based on principles of social cognitive theory, which argues for the important role of cognitive control systems in the acquisition of behavioural proficiencies[29]. The weight loss program is divided into three phases: an intensive, a transition and a maintenance phase in concordance with Messier et al[22]. The main goal of the first phase is to heighten awareness of the importance of and need for changing eating habits. In this phase the ability to read and understand the diversity of labels in food products will be enhanced, and the patient will set goals he believes he can achieve. In the transition phase, problems the patient encounters will be discussed and self-insight will be enhanced concerning the choices that can be made when buying food. Goal in this phase is to prevent relapse. Finally, in the maintenance phase the main objective is to maintain the achieved weight loss and to preserve the motivation to keep on going with the healthy eating habits. Adherence to the intervention is based on attendance at scheduled sessions.

In addition to the combination program (exercise and weight loss), patients receive a manual consisting of written information that focuses on health education, including topics about the medical background of OA, OA treatments, and coping with chronic pain.

### Sample size

Considering the calculation of the sample size, the study of Messier[22] is used as a reference. In this study Messier showed that a combination of exercise and weight reduction in patients with OA of the knee led to a significant improvement (α < 0.05) on the primary outcome measure of self-reported physical function. In order to find an analogous improvement of self-reported physical function of approximately 25% between the first (T0) and last measurements (T2) in patients with OA of the hip, a minimum of 20 patients is needed. This number is based on a power (1-B) of 0.80 and a significance level of 5% (two-sided). When a dropout rate of 20% is taken into account, at least 25 participants have to be included.

### Outcome measurements

At baseline (T0), information is gathered about the patients' demographics (educational level, marital status, family composition) and comorbidities as well as about medication and supplemental use.

### Primary outcome measurement

*The Western Ontario and McMaster Universities Osteoarthritis Index (WOMAC)*: self-reported physical functioning is the primary outcome measure, to be measured with the physical function subscale of the Dutch version of the WOMAC (Dutch-WOMAC)[30,31]. The WOMAC Index is a disease-specific measure of health status and is widely used and recommended in OA research. The validity, reliability and responsiveness of this measure have been demonstrated in an extensive range of studies[32]. The Dutch version of the WOMAC has also been considered valid, reliable and reproducible[31]. The Dutch WOMAC consists of three dimensions: pain (5 items), stiffness (2 items) and physical functioning (17 items). Responses on the 24 items are given on a 5-point Likert scale. All scores will be recoded into a 100-point scale, indicating a score of 0 as the worst possible health condition and 100 the best possible health score.

### Secondary Outcome measurements

To add information about the potential effectiveness of the intervention, participants will be assessed using a range of standardised, self-report measures that include:

1. *Western Ontario and McMaster Universities Osteoarthritis Index (WOMAC)*: the other two dimensions of the Dutch WOMAC, pain and stiffness.

2. *Short Form Health Survey (SF-36)*: the SF-36 measures health-related quality of life and is considered to be valid, reliable and reproducible[33]. The SF-36 is composed of 36 questions, organised into 8 multi-items scales: physical functioning, role limitations due to physical health problems, bodily pain, general health perceptions, vitality, social functioning, role limitations due to emotional problems and general mental health[33].

3. *Short QUestionnaire to ASsess Health enhancing physical activity (SQUASH*): The SQUASH is designed to give an indication of habitual activity level. The SQUASH consists of 6 main questions and is subdivided in 4 categories: (A) commuting activities, (B) leisure-time activities, (C) household activities, and (D) activities at work and school. With the help of the Ainsworth compendium of physical activities[34], the SQUASH subdivides activities into three intensity categories for adults and for older adults (up to age 55 and older). These intensity categories are determined by MET values. MET stands for metabolic equivalent and is defined as 'the ratio of the work metabolic rate to the resting metabolic rate'. For adults, intensity of activities with a MET-value between 2 and < 4 was classified as light, between 4 and < 6.5 as moderate, and ≥ 6.5 as vigorous. For older adults, intensity of activities between 2 and < 3 MET was classified as light, between 3 and < 5 MET as moderate, and ≥ 5 MET as vigorous.

Activities with a MET-value lower than 2 will not be analysed because they are considered to contribute negligibly to habitual activity level. The SQUASH is structured in such a way that it is also possible to assess compliance with physical activity guidelines. The SQUASH is proven to be a fairly reliable and reasonably valid questionnaire[35]. The measurement properties of the SQUASH have been assessed in a population of adults, where it showed an overall reproducibility of 0.58 (95%-CI 0.36–0.74). The relative validity in this study was 0.45 (95%-CI 0.17–0.66) [35]. In a population of overweight people[36] and of people after total hip arthroplasty[37], the Squash was validated with use of an accelerometer with a correlation of 0.40 (p = 0.05) and 0.67 (p = 0.01) respectively. Furthermore, Spearman's correlation coefficient for overall reliability in the overweight study was not applicable, but the hip arthroplasty study showed a value of 0.57 (95%-CI 0.35–0.73)[37].

Patients will also be evaluated using objective measurements, which include:

1. *6-Minute Walk Test (6 MWT)*. The 6 MWT is a functional walking test developed to measure functional status[38]. The test provides information about gait speed and functional and endurance capacity. The primary outcome is the total distance walked. The 6 MWT is considered a reliable test [39,40].

2. *20-Meter Walk Test (20 MWT)*. The 20 MWT is a short, safe test used to measure gait speed like the 10 MWT[41,42]. Patients walk indoors on a 20-m long track, and the time spent to complete the walk (in seconds) will be measured. Time recording will be accomplished with electronic timing equipment by means of photocell gates (HL 2–31 Photocell, Tagheuer, la Chaux-de-Fond, Switzerland).

3. *Weight and fat-free mass assessment*. The amount of lost weight and the amount of fat-free mass can give an indication of improvement of the overweight problem. Weight will be measured with a calibrated scale, always performed by the same dietician. The fat-free mass measurement will be assessed by a hand-held impedance analyser (Omron Body Fat Monitor, model BF 306). It is concluded that the Omron BF 306 body fat monitor yielded results close to the DEXA Body Fat%[43].

4. *Compliance with the program*. Compliance will be registered by AHCRR diet and exercise session attendance. This attendance will be assessed by dividing number of exercise sessions participants actually attended by the number of sessions participants were asked to attend, multiplied by 100%.

**Figure 2:**
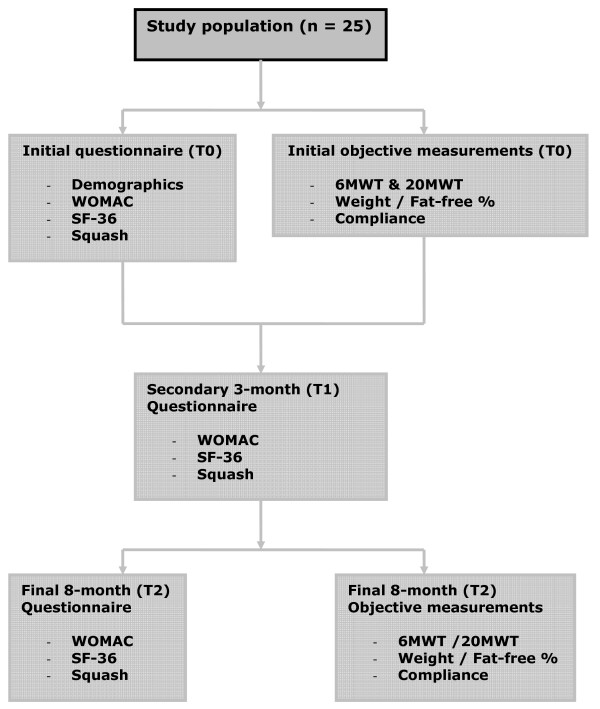
**Study design and assessment points**. T0 = start of the combination program, T1 = 3 months, T2 = 8 months, end of the program.

The first measurement will take place before the combination program begins (T0). The second measurement (T1) will take place at the beginning of the exercise group portion of the combination program after 3 months, and the third measurement (T2) at the end of the combination program after 8 months (see figure 2).

### Statistical analysis

All statistical analyses will be computed using the Statistical Package for the Social Sciences (SPSS, Inc., Version 16.0, 2007, Chicago). Descriptive statistics will be used to describe the group. Changes in response outcomes from measurement points T0 to T2 will be assessed with the GLM ANOVA repeated measurements analyses. Changes in outcomes between measurement points T0 and T2 (pre- and post-measurement) will be analysed with a paired samples T-test. For all test procedures, a probability value of less than 0.05 will be considered as statistically significant.

### Time frame

This study has an 18-month time frame. It is anticipated that identification of potential study participants and recruitment will commence in January 2009. Data analysis will be performed in February 2010 and the final report will be drafted afterwards.

## Discussion

The objective of this prospective cohort study is to get a first glimpse of the potential effectiveness of exercise and weight loss on overweight patients suffering from hip OA. If this study indeed demonstrates that the proposed combination program seems to be effective for hip osteoarthritis, it will be followed by a randomised controlled trial (RCT). In this RCT the effectiveness of the combination program will be investigated in a more controlled setting and will also include a closer look at the cost effectiveness of the combination program. Potential effectiveness of the combination program implies benefits for patients as well as society.

### Patient benefits

A potential benefit for the patient is that the moment of joint replacement can be postponed. Although technical developments have prolonged the lifecycle of hip prostheses, a prosthesis tends to be replaced after a mean of 10 years[44] (what is known as a revision). Revision surgery has greater risks than primary surgery, such as an increased chance of septic loosening. Especially in the case of young people, this is an important reason to postpone a total joint replacement. Conservative therapy (e.g. exercise in combination with weight loss) can therefore be a valuable tool towards accomplishing this[45], and although scientific evidence is lacking, structured exercise and weight loss are already recommended in the clinical setting as a conservative treatment option for patients with OA of the hip[46].

Secondly, regular physical activity can have a positive effect on the general health and fitness of the patient. There is a known dose-response relation between physical activity and health, and according to the recommendations of the ACSM is it important to promote physical activity in older adults that emphasises moderate-intensity aerobic activity and muscle-strengthening activity[26,28]. Regular physical activity has been consistently and reliably linked to a reduction in all-cause mortality, cardiovascular disease and many other debilitating conditions[28]. In addition to the beneficial effects of physical activity on health, regular physical activity also increases older adults' ability to perform their daily activities, thus enhancing their quality of life[47].

In case of weight loss, health benefits are observed in patients with OA in the form of reduced self-reported disability[20] and improved self-reported physical function[20,22]. Published reviews in the obesity literature indicate that obesity impairs health-related quality of life (HRQL) and that higher degrees of obesity are associated with greater impairment[48]. Rejeski[47] pursued this subject in patients with OA, demonstrating that lifestyle modifications like dietary and physical activity behaviours are important interventions for enhancing HRQL [47]. Additionally, weight loss has induced positive improvements in sexual quality of life dimensions[49], which can also be considered as important in the overall rating of quality of life.

### Social benefits

In light of the forecasts of a sharp accumulation of patients with OA, the potential effectiveness of the proposed combination program provides substantial social benefits. This conservative program, considered as lifestyle management, can assist in the approach towards dealing with the large number of people with hip osteoarthritis and most probably reduce the medical costs these patients incur, like physician appointments, medication, outpatient clinical visits and physiotherapy. Eventually, research into the combination of exercise and weight loss in overweight and obese patients suffering from hip OA can provide government agencies and social insurance organisations with evidence to incorporate this kind of therapy for hip osteoarthritis into medical insurance packages. The positive effects of the combination program could end up supporting referral to the program by clinicians caring for people with OA of the hip.

In conclusion, this study will provide highly relevant data on the potential effect of exercise and weight reduction among people suffering from OA of the hip.

## Competing interests

The authors declare that they have no competing interests.

## Authors' contributions

SKB, KvdM and MS originated the idea for the study and will supervise the project. SKB, KvdM, MS, IA and NP were co-applicants of the successful funding proposal. MS and IA contributed to its design, and MS and IA developed the intervention protocol. NP will responsible for the data acquisition and wrote the manuscript. All authors (SKB, IA, MS, KvdM and NP) read and corrected draft versions of the manuscript and approved the final version.

## Pre-publication history

The pre-publication history for this paper can be accessed here:



## References

[B1] Arden N, Nevitt MC (2006). Osteoarthritis: epidemiology. Best Pract Res Clin Rheumatol.

[B2] Brandt K, Kelley W, Harris E, Ruddy S, Sledge C (1985). Osteoarthritis: Clinical patterns and pathology. Textbook of Rheumatology.

[B3] Moskowitz R, McCarthy P (1985). Clinical and laboratory findings in oseoartrthritis. Arthritis and Allied Conditions.

[B4] Wilson MG, Michet CJ, Ilstrup DM, Melton LJ (1990). Idiopathic symptomatic osteoarthritis of the hip and knee: a population-based incidence study. Mayo Clin Proc.

[B5] Volksgezondheid Toekomst Verkenning NKVR. http://www.rivm.nl/vtv/object_document/o1778n18371.html.

[B6] Bemelmans W, Hoogeveen R, Visscher T, Verschuren W, Schuit A Toekomstige ontwikkelingen in overgewicht 7044: inschatting effecten op de volksgezondheid. (Brinkman) B0437044.

[B7] van Baar ME, Assendelft WJJ, Dekker J, Oostendorp RAB, Bijlsma JWJ (1999). Effectiveness of exercise therapy in patients with osteoarthritis of the hip or knee – A systematic review of randomized clinical trials. Arthritis and Rheumatism.

[B8] Rijksinstituut voor Volksgezondheid (RIVM). http://www.rivm.nl/vtv/object_document/o3023n21018.html.

[B9] Corti MC, Rigon C (2003). Epidemiology of osteoarthritis: prevalence, risk factors and functional impact. Aging Clin Exp Res.

[B10] D'Ambrosia RD (2005). Epidemiology of osteoarthritis. Orthopedics.

[B11] Felson DT (1992). Obesity and osteoarthritis of the knee. Bull Rheum Dis.

[B12] National Center for Health Statistics. http://www.cdc.gov/nchs/pressroom/06facts/obesity03_04.htm.

[B13] Seidell JC, Flegal KM (1997). Assessing obesity: classification and epidemiology. Br Med Bull.

[B14] Seidell JC (2002). Prevalence and time trends of obesity in Europe. J Endocrinol Invest.

[B15] Volksgezondheid Toekomst Verkenning NKVR. http://www.rivm.nl/vtv/object_document/o1256n18950.html.

[B16] Persbericht PB08-018. http://www.cbs.nl/NR/rdonlyres/145A65AA-A103-4F15-99CB-3D8187CB2552/0/pb08n018.pdf.

[B17] National Health Center for Health statistics. http://www.cdc.gov/nchs/products/pubs/pubd/hestats/overweight/overwght_adult_03.htm#Table%201.

[B18] O'Reilly SC, Muir KR, Doherty M (1999). Effectiveness of home exercise on pain and disability from osteoarthritis of the knee: a randomised controlled trial. Ann Rheum Dis.

[B19] Roddy E, Doherty M (2006). Changing life-styles and osteoarthritis: what is the evidence?. Best Pract Res Clin Rheumatol.

[B20] Christensen R, Astrup A, Bliddal H (2005). Weight loss: the treatment of choice for knee osteoarthritis? A randomized trial. Osteoarthritis and Cartilage.

[B21] Toda Y, Toda T, Takemura S, Wada T, Morimoto T, Ogawa R (1998). Change in body fat, but not body weight or metabolic correlates of obesity, is related to symptomatic relief of obese patients with knee osteoarthritis after a weight control program. J Rheumatol.

[B22] Messier SP, Loeser RF, Miller GD, Morgan TM, Rejeski WJ, Sevick MA, Ettinger WH, Pahor M, Williamson JD (2004). Exercise and dietary weight loss in overweight and obese older adults with knee osteoarthritis: the Arthritis, Diet, and Activity Promotion Trial. Arthritis Rheum.

[B23] Vignon E, Valat JP, Rossignol M, Avouac B, Rozenberg S, Thoumie P, Avouac J, Nordin M, Hilliquin P (2006). Osteoarthritis of the knee and hip and activity: a systematic international review and synthesis (OASIS). Joint Bone Spine.

[B24] Fransen M, McConnell S, Bell M (2003). Exercise for osteoarthritis of the hip or knee. Cochrane Database Syst Rev.

[B25] Kellgren JH, Lawrence JS (1957). Radiological assessment of osteo-arthrosis. Ann Rheum Dis.

[B26] Haskell WL, Lee IM, Pate RR, Powell KE, Blair SN, Franklin BA (2007). Physical activity and public health: updated recommendation for adults from the American College of Sports Medicine and the American Heart Association. Med Sci Sports Exerc.

[B27] Kemper H, Ooijendijk W, Stiggelbout M, Hildebrandt V, Backx F, Bol E De Nederlandse norm gezond bewegen: verslag van een expertmeeting. Trendrapport bewegen en gezondheid 1998/1999.

[B28] Nelson ME, Rejeski WJ, Blair SN, Duncan PW, Judge JO, King AC (2007). Physical activity and public health in older adults: recommendation from the American College of Sports Medicine and the American Heart Association. Med Sci Sports Exerc.

[B29] Bandura A (1986). Social foundations of thought and action: A social cognitive theory.

[B30] Bellamy N, Buchanan WW, Goldsmith CH, Campbell J, Stitt LW (1988). Validation study of WOMAC: a health status instrument for measuring clinically important patient relevant outcomes to antirheumatic drug therapy in patients with osteoarthritis of the hip or knee. J Rheumatol.

[B31] Roorda LD, Jones CA, Waltz M, Lankhorst GJ, Bouter LM, Eijken JW van der, Willems WJ, Heyligers IC, Voaklander DC, Kelly KD (2004). Satisfactory cross cultural equivalence of the Dutch WOMAC in patients with hip osteoarthritis waiting for arthroplasty. Ann Rheum Dis.

[B32] McConnell S, Kolopack P, Davis AM (2001). The Western Ontario and McMaster Universities Osteoarthritis Index (WOMAC): a review of its utility and measurement properties. Arthritis Rheum.

[B33] Aaronson NK, Muller M, Cohen PD, Essink-Bot ML, Fekkes M, Sanderman R, Sprangers MA, te Velde A, Verrips E (1998). Translation, validation, and norming of the Dutch language version of the SF-36 Health Survey in community and chronic disease populations. J Clin Epidemiol.

[B34] Ainsworth BE, Haskell WL, Leon AS, Jacobs DR, Montoye HJ, Sallis JF, Paffenbarger RS (1993). Compendium of physical activities: classification of energy costs of human physical activities. Med Sci Sports Exerc.

[B35] Wendel-Vos GC, Schuit AJ, Saris WH, Kromhout D (2003). Reproducibility and relative validity of the short questionnaire to assess health-enhancing physical activity. J Clin Epidemiol.

[B36] Kwak L, Kremers S, Brug J, Van Baak M (2007). Measuring physical activity in field studies: Comparison of a questionnaire, 24-hour recall and an accelerometer. European journal of sport science.

[B37] Wagenmakers R, Akker-Scheek I van den, Groothoff JW, Zijlstra W, Bulstra SK, Kootstra JW, Wendel-Vos GC, van Raaij JJ, Stevens M (2008). Reliability and validity of the short questionnaire to assess health-enhancing physical activity (SQUASH) in patients after total hip arthroplasty. BMC Musculoskelet Disord.

[B38] Enright PL (2003). The six-minute walk test. Respir Care.

[B39] Bassey EJ, Fentem PH, MacDonald IC, Scriven PM (1976). Self-paced walking as a method for exercise testing in elderly and young men. Clin Sci Mol Med Suppl.

[B40] Singh S (1992). The use of field walking tests for assessment of functional capacity in patients with chronic airways obstruction. Physiotherapy.

[B41] Juhakoski R, Tenhonen S, Anttonen T, Kauppinen T, Arokoski JP (2008). Factors affecting self-reported pain and physical function in patients with hip osteoarthritis. Arch Phys Med Rehabil.

[B42] Strand LI, Wie SL (1999). The Sock Test for evaluating activity limitation in patients with musculoskeletal pain. Phys Ther.

[B43] Lintsi M, Kaarma H, Kull I (2004). Comparison of hand-to-hand bioimpedance and anthropometry equations versus dual-energy X-ray absorptiometry for the assessment of body fat percentage in 17–18-year-old conscripts. Clin Physiol Funct Imaging.

[B44] Havelin LI, Engesaeter LB, Espehaug B, Furnes O, Lie SA, Vollset SE (2000). The Norwegian Arthroplasty Register: 11 years and 73,000 arthroplasties. Acta Orthop Scand.

[B45] Harrysson OL, Robertsson O, Nayfeh JF (2004). Higher cumulative revision rate of knee arthroplasties in younger patients with osteoarthritis. Clin Orthop Relat Res.

[B46] Lane NE (2007). Clinical practice. Osteoarthritis of the hip. N Engl J Med.

[B47] Rejeski WJ, Focht BC, Messier SP, Morgan T, Pahor M, Penninx B (2002). Obese, older adults with knee osteoarthritis: weight loss, exercise, and quality of life. Health Psychol.

[B48] Fontaine KR, Barofsky I (2001). Obesity and health-related quality of life. Obes Rev.

[B49] Kolotkin RL, Binks M, Crosby RD, Ostbye T, Mitchell JE, Hartley G (2008). Improvements in sexual quality of life after moderate weight loss. Int J Impot Res.

